# CXCL4 deficiency limits M4 macrophage infiltration and attenuates hyperoxia-induced lung injury

**DOI:** 10.1186/s10020-024-01043-y

**Published:** 2024-12-20

**Authors:** Bingrui Yu, Siyuan Jia, Yu Chen, Rong Guan, Shuyu Chen, Wanwen Tang, Tianping Bao, Zhaofang Tian

**Affiliations:** Department of Neonatology, The Affiliated Huaian No.1 People’s Hospital of Nanjing Medical University, No.1 Western Huanghe Road, Huai’an, Jiangsu 223300 China

**Keywords:** BPD, CXCL4, M4 macrophages, Hyperoxia, Lung injury, Neonatal mice

## Abstract

**Background:**

Bronchopulmonary dysplasia (BPD), a chronic lung disease prevalent among premature infants, significantly impacts lifelong respiratory health. Macrophages, as key components of the innate immune system, play a role in lung tissue inflammation and injury, exhibiting diverse and dynamic functionalities. The M4 macrophage, a distinctive subtype primarily triggered by chemokine (C-X-C motif) ligand 4 (CXCL4), has been implicated in pulmonary inflammatory and fibrotic processes. Nonetheless, its contribution to the pathophysiology of BPD remains uncertain.

**Objective:**

This study aimed to elucidate the involvement of CXCL4 in hyperoxia-induced neonatal lung injury and fibrosis, with a particular focus on its influence on M4 macrophages.

**Methods:**

A BPD model in neonatal mice was established through continuous exposure to 95% O_2_ for 7 days. Comparative analyses of lung damage and subsequent regeneration were conducted between wild-type (WT) and CXCL4 knockout (KO) mice. Lung tissue inflammation and fibrosis were assessed using histological and immunofluorescence staining, enzyme-linked immunosorbent assay, Western blot, and real-time quantitative polymerase chain reaction. Differentiation of M0 and M4 macrophages was performed in vitro using macrophage colony-stimulating factor and CXCL4, while expressions of S100A8 and MMP7, along with migration assays, were evaluated.

**Results:**

Elevated CXCL4 levels and M4 macrophage activation were identified in the lung tissue of BPD model mice. CXCL4 deficiency conferred protection to alveolar type 2 epithelial cells, reduced sphingosine-1-phosphate metabolic activity, mitigated pulmonary fibrosis, and limited M4 macrophage progression. This deletion further enhanced lung matrix remodeling during recovery. In vitro, CXCL4 promoted M4 macrophage differentiation and increased macrophage migration via chemokine (C-C motif) receptor 1.

**Conclusion:**

CXCL4 contributes to hyperoxia-induced lung injury and fibrosis through modulation of cytokine release, alveolar cell proliferation, lipid metabolism, and the regulation of macrophage phenotype and function.

## Introduction

Bronchopulmonary dysplasia (BPD), a chronic lung disease, predominantly impacts premature infants with low birth weight. Significant advancements in neonatal care have enhanced survival rates; however, BPD still affects nearly 40% of infants born before 29 weeks of gestation (Stoll et al. 2015). The primary cause of BPD is underdeveloped lungs, with prenatal and postnatal factors contributing to inflammatory responses that disrupt normal lung growth and repair mechanisms (Baraldi and Filippone 2007). Central to BPD pathogenesis is inflammation driven by macrophage activation (Hirani et al. 2023). The immature state of alveolar macrophages at birth predisposes newborns to lung conditions (Honda et al. 2022).

Macrophage polarization plays a key role in the progression of various diseases. These immune cells exhibit a spectrum of phenotypes, with the classical (M1) and alternative (M2) subtypes marking the extremes (Paolicelli et al. 2022). M2 macrophages are categorized into M2a, M2b, M2c, and M2d subtypes, each performing distinct biological roles (Martinez et al. 2008). A range of macrophage phenotypes has been observed within atherosclerotic tissues, including hemoglobin-associated macrophages, hemoglobin-activated macrophages, Mox (induced by oxidized phospholipids), and M4 macrophages (Ni et al. 2023; Parisi et al. 2018; Kalish et al. 2017). M4 macrophages represent a unique subtype predominantly triggered by chemokine (C-X-C motif) ligand 4 (CXCL4), leading to monocyte differentiation. The markers CD68, S100 calcium-binding protein A8 (S100A8), and matrix metalloproteinase 7 (MMP7) are indicative of the M4 phenotype. Initially identified within coronary atherosclerotic plaques, M4 macrophages have been linked to plaque instability (Domschke and Gleissner 2019). Their presence has since been noted in leprosy-associated skin lesions and myocardial infarction sites (Sousa et al. 2018; Lindsey et al. 2019).

CXCL4, known as platelet factor 4 (PF4), plays a significant role in the modulation of macrophage polarization (Hoeft et al. 2023). Notably, CXCL4 promotes the M1 macrophage phenotype while suppressing the M2 phenotype. This chemokine participates in inflammatory responses, innate immunity, and bacterial eradication (Yue et al. 2018). By binding to bacteria, it triggers neutrophil activation through diverse intracellular signaling mechanisms (Schrottmaier et al. 2024). The unique involvement of CXCL4 in macrophage polarization and its links to inflammatory pathologies underscore its potential for therapeutic intervention.

Exposure to elevated O_2_ levels is integral to BPD pathogenesis, particularly in preterm infants who require O_2_ supplementation due to underdeveloped lungs incapable of adequate gas exchange (Larson-Casey and Carter 2022). Rodent models remain optimal for examining hyperoxia-induced pulmonary damage and assessing therapeutic interventions (Giusto et al. 2021). The involvement of various macrophage subtypes, notably M4 macrophages, in hyperoxia-related lung damage is currently being explored (Hall et al. 2006). Hyperoxic conditions can exacerbate existing pulmonary injuries (Singer et al. 2021). Prior studies employed tandem mass tags proteomic analysis to compare lung proteomes between neonatal mice of both sexes under hyperoxic exposure, revealing CXCL4 as specifically altered in male neonates (Cheng et al. 2020).

This study investigated CXCL4’s contribution to M4 macrophage infiltration and its influence on hyperoxia-induced lung pathology. Deciphering these mechanisms may yield critical insights into hyperoxia-induced lung injury pathogenesis and uncover potential therapeutic targets.

## Materials and methods

### Animal model

Wild-type (WT) C57BL/6J and CXCL4 knockout (KO) mice were obtained from Saiye Biotechnology, Suzhou, and maintained under specific pathogen-free conditions. Procedures adhered to the Guide for the Care and Use of Laboratory Animals by the National Institutes of Health and received approval from the Ethics Committee of The Affiliated Huaian No. 1 People’s Hospital, Nanjing Medical University. Neonatal mice (within 24 h) were randomly divided into four experimental groups: WT mice subjected to normoxia (NOX; 21% O₂), WT mice subjected to hyperoxia (HYX; 95% O₂), CXCL4 KO mice exposed to NOX, and CXCL4 KO mice exposed to HYX. Each group comprised 6 mice. The animals were placed in a custom airtight chamber, with O₂ concentration continuously monitored via an O₂ analyzer. Normoxia exposure was set at 21% O₂ for 7 days, while hyperoxia was maintained at 95% O₂ for 7 days, with daily calibration to ensure consistent O₂ levels. Food and water were provided without restriction. Following the exposure period, euthanasia was performed using CO₂ overdose followed by exsanguination. Lung perfusion with phosphate-buffered saline (PBS) was conducted through the right ventricle, and lung tissues were subsequently collected.

### Hematoxylin and eosin staining

Lung tissues were collected and fixed in 4% paraformaldehyde (PFA) (P6148, Sigma-Aldrich, USA) before being embedded. Hematoxylin and eosin (H&E) staining was applied to the sections, and digital imaging was performed using an Infinity1 camera (Lumenera Corporation, USA) connected to an Axio ImagerZ1 light microscope (Zeiss Microscopy, Germany). The radial alveolar count (RAC) was assessed following the method by Emery and Mithal (Guzmán-Navarro et al. 2021), defined as the number of closed alveoli intersected by a line drawn perpendicularly from the terminal bronchiole to the nearest pleura. Septal thickness measurements were obtained from H&E-stained sections. Alveolarization levels were quantified through mean cord length (L_m_) and alveolar surface area. L_m_, representing the distance between airspace walls, was calculated by counting wall intersections with an 84-line array, each approximately 24 μm in length. Tissue volume density (VD_T_) was analyzed using a 10 × 10 grid (side length ~ 29 μm). The SA was computed using the equation SA = 4 × VDT × lung volume / L_m_.

### Flow cytometry

Lung tissues were collected and cut into smaller fragments using sterile scissors, followed by enzymatic digestion in a solution of collagenase D (11088858001, Roche, Germany) (2 mg/mL) and DNase I (10104159001, Roche, Germany) (0.1 mg/mL) at 37 °C for 45 min. Cell counts were performed with a hemocytometer, and the cells were subsequently resuspended. Staining was conducted with antibodies specific to F4/80 (MCA497G, Bio-Rad, USA), S100A8 (MAB4570, R&D Systems, USA), and MMP7 (ab51072, Abcam, UK) for 30 min at 4 °C under dark conditions. Following a washing step, cells were fixed in 1% PFA (158127, Sigma-Aldrich, USA) and subjected to analysis on a BD LSRFortessa flow cytometer.

### Quantitative real-time polymerase chain reaction

Following RNA extraction and cDNA synthesis, quantitative real-time polymerase chain reaction (qRT-PCR) was performed with SYBR Green Master Mix (A25742, Thermo Fisher, USA) on a CFX96 Real-Time PCR System (Bio-Rad). CXCL4 expression was assessed using designated primers (forward: 5’-AGTGGCTGTCATCGCTTTG-3’, reverse: 5’-GGTGGCAGAGAGGAAGAGC-3’). For S100A8, primer sequences included (forward: 5’-GCCCTCTACAAGAATGACTTCAAG-3’, reverse: 5’-ATCACCATCGCAAGGAACTCC-3’), while MMP7 expression utilized primers (forward: 5’-CTGCCACTGTCCCAGGAAG-3’, reverse: 5’-GGGAGAGTTTTCCAGTCATGG-3’). GAPDH expression was measured with specific primers (forward: 5’-AGGTCGGTGTGAACGGATTTG-3’, reverse: 5’-GGGGTCGTTGATGGCAACA-3’).

### Enzyme-linked immunosorbent assay

Samples were homogenized in ice-cold radioimmunoprecipitation assay buffer using a tissue homogenizer (T10, IKA, Germany). Following homogenization, centrifugation at 14,000 g for 20 min at 4 °C was performed to remove debris. CXCL4 concentrations were quantified with a mouse CXCL4/PF-4 ELISA kit (MCX400, R&D Systems, USA). Levels of MMP7 and S100A8 in cell culture supernatants were determined using ELISA kits (MMP7: ab100607, Abcam, UK; S100A8: ab205715, Abcam, UK). For the assay, 96-well plates were coated with the capture antibody and left at room temperature overnight. Plates were then washed with 0.05% Tween 20 in PBS and blocked for 1 h at room temperature using 1% bovine serum albumin (BSA) in PBS. Samples and standards were subsequently added to the wells. Following an additional wash, the detection antibody was applied and incubated for 2 h, followed by a 30-minute incubation with streptavidin-horseradish peroxidase. The plates underwent further washing before development with 3,3’,5,5’-tetramethylbenzidine substrate solution (34021, Thermo Fisher Scientific, USA). The reaction was terminated with 2 N sulfuric acid (258105, Sigma-Aldrich, USA), and absorbance was measured at 450 nm using a microplate reader (ELx808, BioTek, USA).

### Immunofluorescence (IF) staining

Lung tissue sections were deparaffinized, rehydrated, and subjected to antigen retrieval using pH 6.0 citrate buffer. Blocking was performed with 5% BSA (A9647, Sigma-Aldrich, USA), followed by incubation with terminal deoxynucleotidyl transferase dUTP nick-end labeling (TUNEL) reagent (C1088-50, Beyotime, China) and primary antibodies targeting F4/80 (MCA497G, Bio-Rad, USA), S100A8 (MAB4570, R&D Systems, USA), 4’,6-diamidino-2-phenylindole (D1306, Thermo Fisher, USA), MMP7 (ab51072, Abcam, UK), and surfactant protein C (SFTPC) (A11012, Thermo Fisher, USA). Fluorophore-labeled secondary antibodies (Alexa Fluor 488, A11008; Alexa Fluor 594, A11012, Thermo Fisher) were applied. Image acquisition was conducted using a confocal microscope (Leica, TCS SP8).

### Western blotting

Lung tissues were homogenized in ice-cold RIPA buffer (89900, Thermo Fisher, USA) using a tissue homogenizer (T10, IKA, Germany). Homogenates underwent centrifugation at 14,000 g for 20 min at 4 °C to eliminate debris, and supernatants were collected. Protein samples (20 µg per lane) were resolved on 4–20% Mini-PROTEAN TGX gels (4561093, Bio-Rad, USA) and transferred onto membranes (IPVH00010, Millipore, USA) via a wet transfer system (Trans-Blot Turbo, Bio-Rad, USA). The primary antibodies used included CXCL4 (ab134087, Abcam, UK), TNF-α (3707, Cell Signaling, USA), IL-6 (12912, Cell Signaling, USA), MMP7 (ab51072, Abcam, UK), S100A8 (MAB4570, R&D Systems, USA), GAPDH (5174, Cell Signaling, USA), SPHK1 (12071, Cell Signaling, USA), SPHK2 (ab107146, Abcam, UK), SPT2 (sc-365859, Santa Cruz Biotechnology, USA), S1PL (PA5-53002, Thermo Fisher, USA), p-SMAD2 (3108, Cell Signaling, USA), and SMAD2 (5339, Cell Signaling, USA), with GAPDH serving as the loading control. After primary antibody incubation, membranes were rinsed with TBST and exposed to HRP-conjugated secondary antibodies (31460, Thermo Fisher, USA). Protein bands were visualized using a chemiluminescence imaging system (ChemiDoc XRS+, Bio-Rad, USA).

### Verhoeff-van gieson staining for elastic fibers

Elastic fibers in lung tissue were visualized using the Verhoeff-Van Gieson staining technique (Verhoeff Elastic Stain Kit, HT25, Sigma-Aldrich, USA). Tissue sections underwent deparaffinization and rehydration before being treated with Verhoeff stain, followed by counterstaining with Van Gieson’s solution. Imaging was performed, and quantification of elastic fiber content was conducted through image analysis software. The proportion of the area covered by elastic fibers was determined from multiple randomly selected fields per section.

### Quantifying collagen content

Collagen deposition in lung tissue was assessed using Sirius Red staining (365548, Sigma-Aldrich, USA). Tissue sections underwent deparaffinization, rehydration through a graded series of alcohols, and were then incubated with Sirius Red solution for 1 h. Following staining, sections were rinsed in 0.5% acetic acid, dehydrated with alcohols, cleared in xylene, and mounted with DPX Mountant (Sigma-Aldrich, USA). Polarized light microscopy was used to capture images of the stained sections. Quantification of collagen content was performed using image analysis software, calculating the percentage of lung tissue area occupied by collagen fibers across multiple fields.

### Murine primary macrophage model

Murine primary bone marrow-derived macrophages were generated following established protocols (Marim et al. 2010). Fresh bone marrow cells were harvested from the femurs and tibias of WT C57BL/6J mice and cultured in Dulbecco’s Modified Eagle’s medium (DMEM) supplemented with 10% heat-inactivated FBS and either macrophage colony-stimulating factor (M-CSF) (100 ng/mL, 216-MC-050, R&D Systems, USA) or CXCL4 (4 µM, ab188288, Abcam, UK) to differentiate into M0 or M4 macrophages over a 7-day period. Cells were seeded in 8 ml of medium in 100 mm non-tissue culture dishes (Thermo Fisher Scientific, USA), with an additional 5 ml of medium introduced on day 5. Cell detachment from the dish surface was performed using a gentle scraping technique. The collected macrophages were subsequently counted and resuspended in FBS-free medium.

The purity of murine primary bone marrow-derived macrophages was validated through flow cytometry using F4/80 and CD11b antibodies (eBioscience). To block Fc receptors, cells were incubated on ice with FBS for 15 min, followed by incubation with primary antibodies for 30 min under the same conditions. Cells were then fixed in 2% formaldehyde and analyzed with a BD LSRII flow cytometer, with data processed via FlowJo software. FACS analysis indicated that bone marrow-derived macrophages consistently exhibited > 95% positivity for CD11b and F4/80.

The CCR1 inhibitor BX471 (1 nM) was sourced from MedChemExpress (New Jersey, USA), with J-113863, a known CCR1 antagonist, used as a positive control (Amat et al. 2006).

### Migration assay

The migratory capacity of murine primary macrophages was evaluated using a Transwell migration assay. Macrophages (1 × 10^5^/ml) were placed in the upper chamber of Transwell inserts (3422, Corning, USA) with serum-free medium, while the lower chamber contained medium supplemented with 10% FBS serving as a chemoattractant. Following a 24-hour incubation at 37 °C, migrated macrophages on the lower membrane surface were stained with Crystal Violet (C3886, Sigma-Aldrich, USA) and visualized under an Olympus BX53 microscope (Japan).

### Statistical analysis

Experiments were conducted a minimum of three times. Data were presented as mean ± standard deviation (SD). Statistical analysis was performed using GraphPad Prism (GraphPad Software, USA). Group comparisons were evaluated through one-way ANOVA, with Tukey’s post hoc test applied for multiple comparisons. Statistical significance was defined as *p <* 0.05.

## Results

### M4 macrophages were activated and CXCL4 was upregulated in BPD

**Fig. 1:**
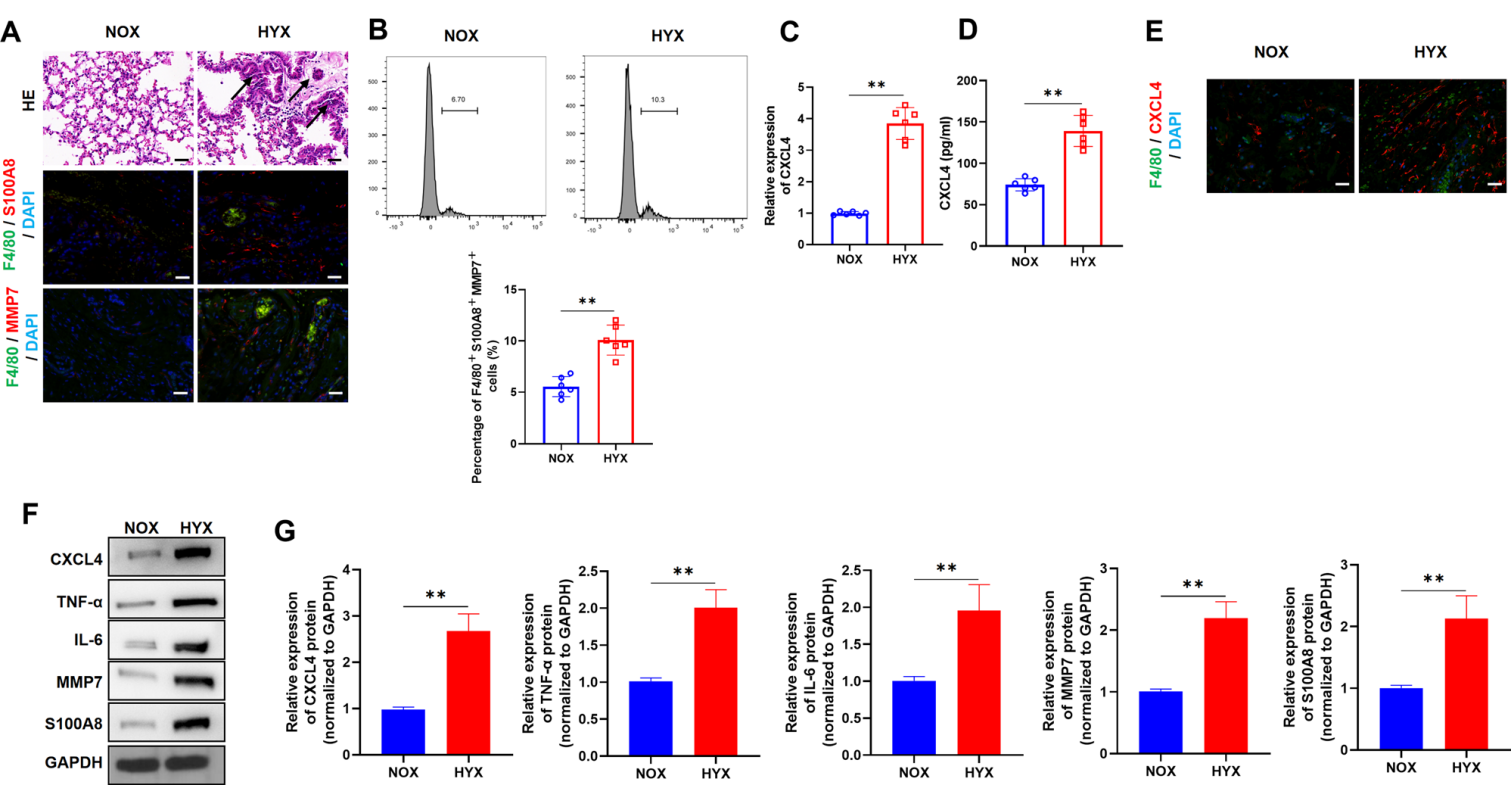
M4 macrophages are activated and CXCL4 is upregulated in BPD. (**A**) Representative images of H&E staining (arrow represented the pathological structural change in the lung tissues of mice) and IF for F4/80 (a macrophage marker), S100A8, MMP7, and DAPI in lung tissues from NOX (21% O2) and HYX (95% O2) groups (*n* = 6). (**B**) Flow cytometric analysis showing the percentage of F4/80^+^S100A8^+^MMP7^+^ cells in NOX and HYX groups (*n* = 6). (**C**-**D**) qRT-PCR analysis and ELISA quantification of CXCL4 expression levels (*n* = 6). (**E**) IF staining for F4/80 and CXCL4 in lung tissues (*n* = 6). (**F**-**G**) Western blot analysis of CXCL4, TNF-α, IL-6, MMP7, S100A8, and GAPDH in lung tissues from NOX and HYX groups (*n* = 6)

A BPD mouse model was employed to examine the impact of hyperoxia on lung development. H&E staining revealed that in normoxic (NOX; 21% O_2_) conditions, the alveolar structure was well-formed and uniform. In contrast, after 7 days of hyperoxic exposure (HYX; 95% O_2_), the lung tissue exhibited significant pathological changes, including disorganized lung architecture, thickened pulmonary septa, alveolar fusion and enlargement, and a reduced alveolar count. These alterations were further confirmed by immunofluorescence (IF) staining, which revealed an increase in F4/80-positive cells (M4 macrophages), alongside elevated levels of S100A8 and MMP7, markers indicative of M4 macrophage activation, in the HYX group (Fig. 1A). The proportion of F4/80 + S100A8 + MMP7 + cells was notably higher under hyperoxic conditions (Fig. 1B). qRT-PCR, ELISA, and IF analysis consistently indicated a substantial upregulation of CXCL4, a chemokine involved in immune cell recruitment, in the HYX group (Fig. 1C-E). As previously noted, the hyperoxia-induced inflammatory response and subsequent tissue damage were key factors in the pathogenesis of BPD in the developing lung (Shahzad et al. 2022). Western blot analysis further supported these observations, showing elevated levels of CXCL4 protein, along with increased expression of other inflammatory markers, including tumor necrosis factor-alpha (TNF-α), immunoglobulin (IL)-6, MMP7, and S100A8, in the HYX group (Fig. 1F-G). Taken together, hyperoxia disrupts normal lung development and induces a harmful inflammatory response, characterized by CXCL4 upregulation.

### CXCL4 deficiency alleviates hyperoxia-induced alveolar epithelial type 2 cell injury and suppresses sphingosine-1-phosphate metabolism

**Fig. 2:**
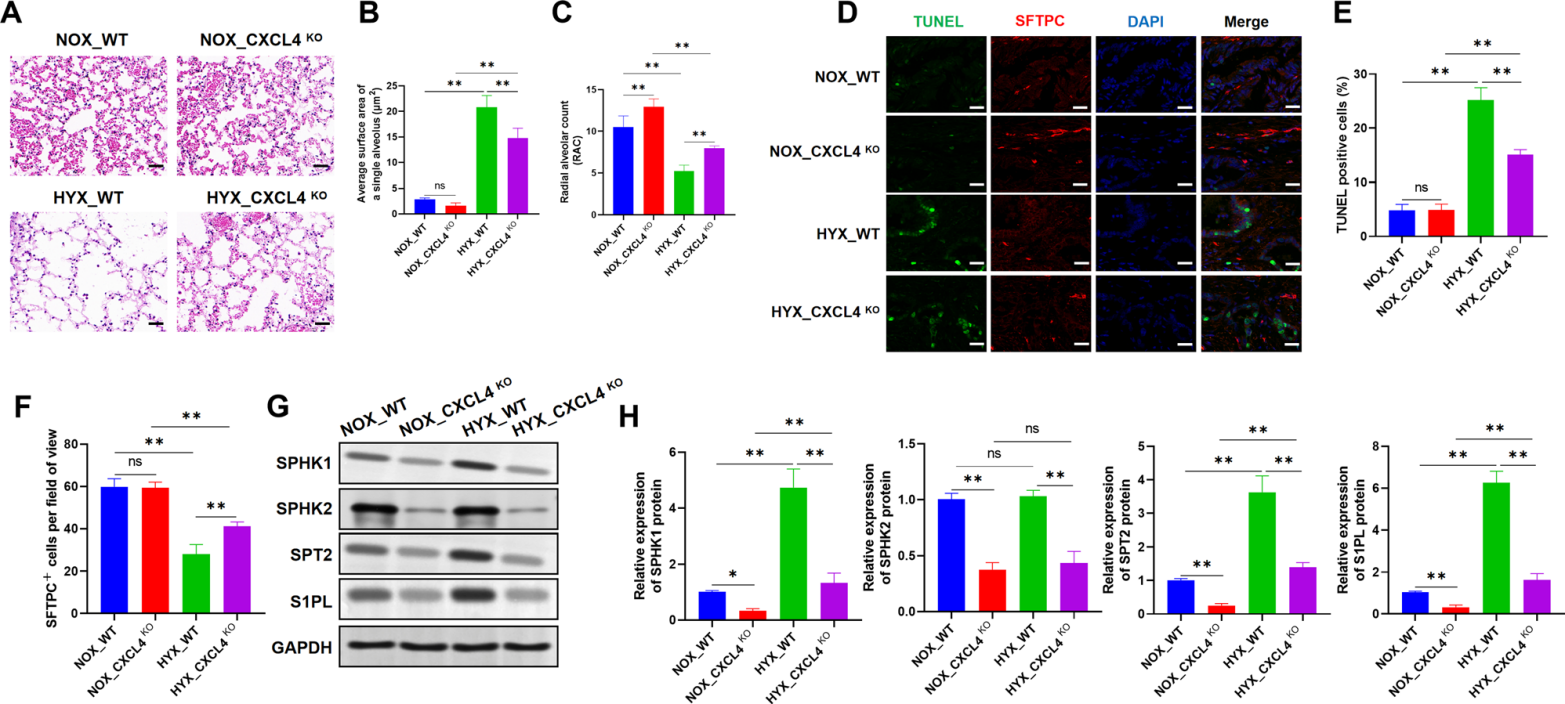
Loss of CXCL 4 alleviates hyperoxia-induced alveolar epithelial type 2 cells (AT2) injury and suppresses S1P metabolism. (**A**) H&E staining of lung tissues from WT and CXCL4 KO mice exposed to NOX (21% O_2_) and HYX (95% O2) conditions for 7 days (*n* = 6). (**B**) Quantification of the average surface area of a single alveolus (µm²) (*n* = 6). (**C**) Radial Alveolar Count (RAC) analysis (*n* = 6). (**D**) Representative images of TUNEL staining (green), SFTPC (red), and DAPI (blue) in lung sections, indicating apoptosis and AT2 cell distribution (*n* = 6). (**E**) Quantification of TUNEL-positive cells (*n* = 6). (**F**) Quantification of SFTPC + cells per field of view (*n* = 6). (**G**-**H**) Western blot analysis of SPHK1, SPHK2, SPT2, and S1PL, genes associated with sphingosine-1-phosphate metabolism (*n* = 6)

WT and CXCL4 KO mice were exposed to normoxia or hyperoxia for 7 days. H&E staining revealed that, compared to HYX_WT mice, alveolar damage was mitigated and pulmonary septal thickening was alleviated in HYX_CXCL4 KO mice (Fig. 2A). The average surface area of a single alveolus increased in HYX_WT mice but was relatively decreased in HYX_CXCL4 KO mice. Furthermore, the alveolar surface area in HYX_CXCL4 KO mice was higher than in NOX_CXCL4 KO mice (Fig. 2B). Likewise, the RAC was reduced in HYX_WT mice, but was relatively increased in HYX_CXCL4 KO mice. The RAC in HYX_CXCL4 KO mice was lower than in NOX_CXCL4 KO mice (Fig. 2C). The number of TUNEL-positive cells, indicative of apoptosis, increased in HYX_WT mice but was relatively decreased in HYX_CXCL4 KO mice. The apoptosis was more significant in HYX_CXCL4 KO mice compared to NOX_CXCL4 KO mice (Fig. 2D-E). The number of SFTPC + cells per field, reflecting type 2 alveolar cells, decreased in HYX_WT mice but was relatively increased in HYX_CXCL4 KO mice. However, the number of SFTPC + cells in HYX_CXCL4 KO mice was lower than in NOX_CXCL4 KO mice (Fig. 2F). To our knowledge, sphingosine-1-phosphate (S1P) is a potent angiogenic factor that stabilizes lung endothelial cell integrity and reduces vascular permeability and alveolar flooding in preclinical animal models of lung injury (Gao et al. 2024). Western blot analysis revealed elevated protein expression of sphingosine kinase 1 (SPHK1), sphingosine kinase 2 (SPHK2), serine palmitoyltransferase 2 (SPT2), and sphingosine 1-phosphate lyase (S1PL)—key regulators in S1P metabolism—in HYX_WT mice, while expression levels were relatively lower in HYX_CXCL4 KO mice. Notably, in HYX_CXCL4 KO mice, protein levels of SPHK1, SPT2, and S1PL were higher compared to NOX_CXCL4 KO mice (Fig. 2G-H). These results suggest that CXCL4 deficiency mitigates hyperoxia-induced type 2 alveolar cell injury and suppresses the expression of S1P metabolism-associated genes under hyperoxic conditions.

### CXCL4 deletion weakens fibrotic lung remodeling

**Fig. 3:**
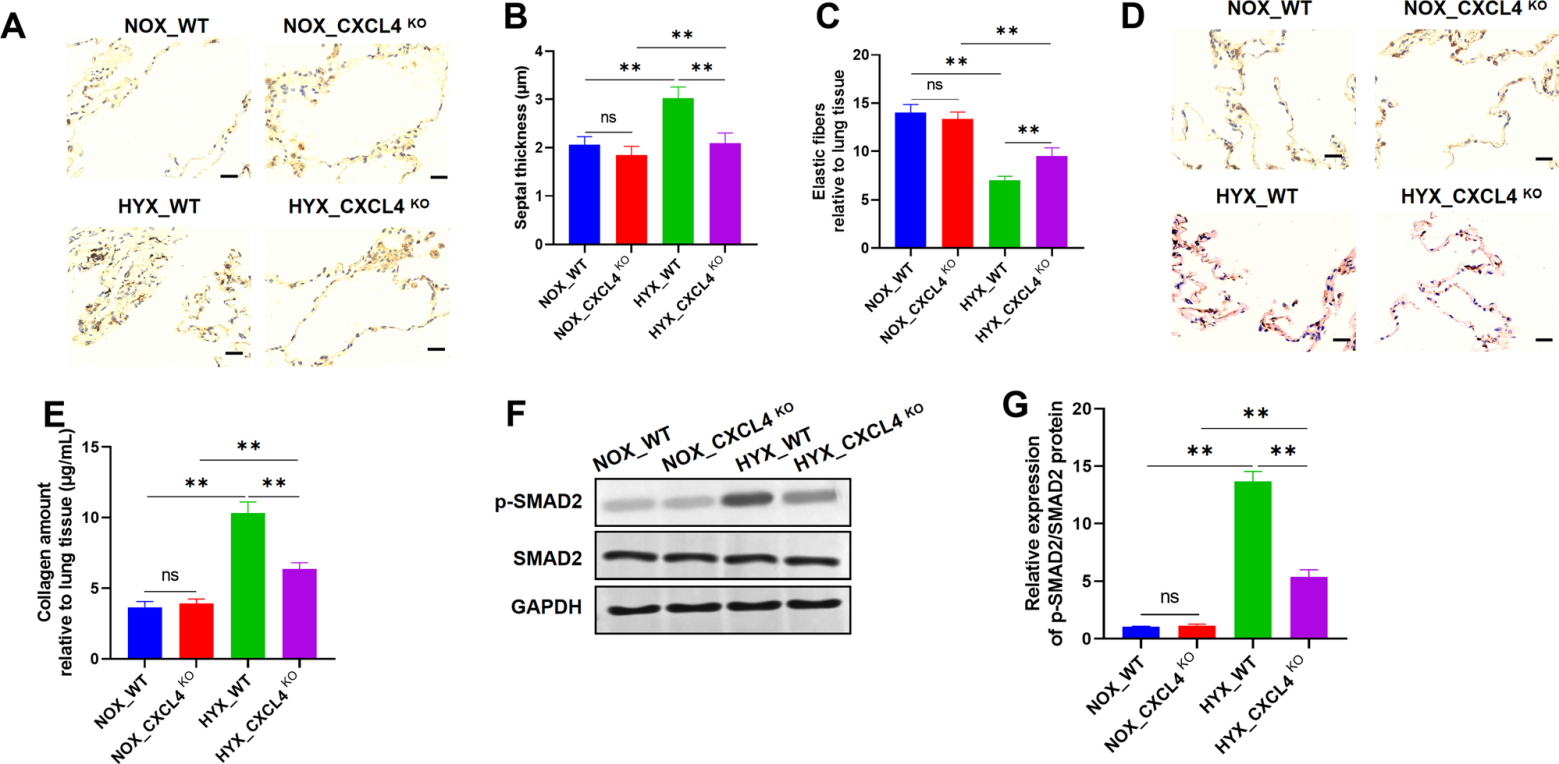
Deletion of CXCL4 weakens fibrotic lung remodeling. (**A**) Representative images of lung tissue sections from WT and CXCL4 KO mice under NOX (21% O_2_) and HYX (95% O_2_) conditions (*n* = 6). (**B**) Measurement of septal thickness (µm) in the NOX_WT, NOX_CXCL4 KO, HYX_WT, and HYX_CXCL4 KO groups (*n* = 6). (**C**) Analysis of elastic fibers relative to lung tissue (*n* = 6). (**D**) Sirius Red staining showing changes in collagen content (*n* = 6). (**E**) Quantification of collagen amount relative to lung tissue (*n* = 6). (**F**) Western blot analysis of phosphorylated SMAD2 (*n* = 6). (**G**) Relative expression levels of p-SMAD2/SMAD2 protein ratio (*n* = 6)

In comparison to NOX_WT mice, septal thickness was increased in HYX_WT mice, whereas it was reduced in HYX_CXCL4 KO mice relative to HYX_WT mice. Notably, the septal thickness in HYX_CXCL4 KO mice remained higher than that in NOX_CXCL4 KO mice (Fig. 3A-B). A similar pattern was observed in the distribution of elastic fibers: their content was lower in HYX_WT mice than in NOX_WT mice, but increased in HYX_CXCL4 KO mice relative to HYX_WT mice. However, the elastic fiber content in HYX_CXCL4 KO mice was still lower than that observed in NOX_CXCL4 KO mice (Fig. 3C). Collagen content was elevated in HYX_WT mice compared to NOX_WT mice but was relatively reduced in HYX_CXCL4 KO mice. Moreover, the collagen in HYX_CXCL4 KO mice was greater than that in NOX_CXCL4 KO mice (Fig. 3D-E). Previous studies have established that phosphorylated SMAD2 (p-SMAD2) plays a critical role in lung remodeling (Pan et al. 2024). Thus, p-SMAD2 levels were further assessed in lung tissues. Western blot analysis revealed that p-SMAD2 protein levels were higher in HYX_WT mice compared to NOX_WT mice, but lower in HYX_CXCL4 KO mice relative to HYX_WT mice. Additionally, the reduced p-SMAD2 levels in HYX_CXCL4 KO mice were still higher than those in NOX_CXCL4 KO mice (Fig. 3F-G). These results suggest that CXCL4 deficiency may attenuate hyperoxia-induced lung injury and associated fibrotic remodeling.

### CXCL4 deletion prevents M4 macrophage progression

**Fig. 4:**
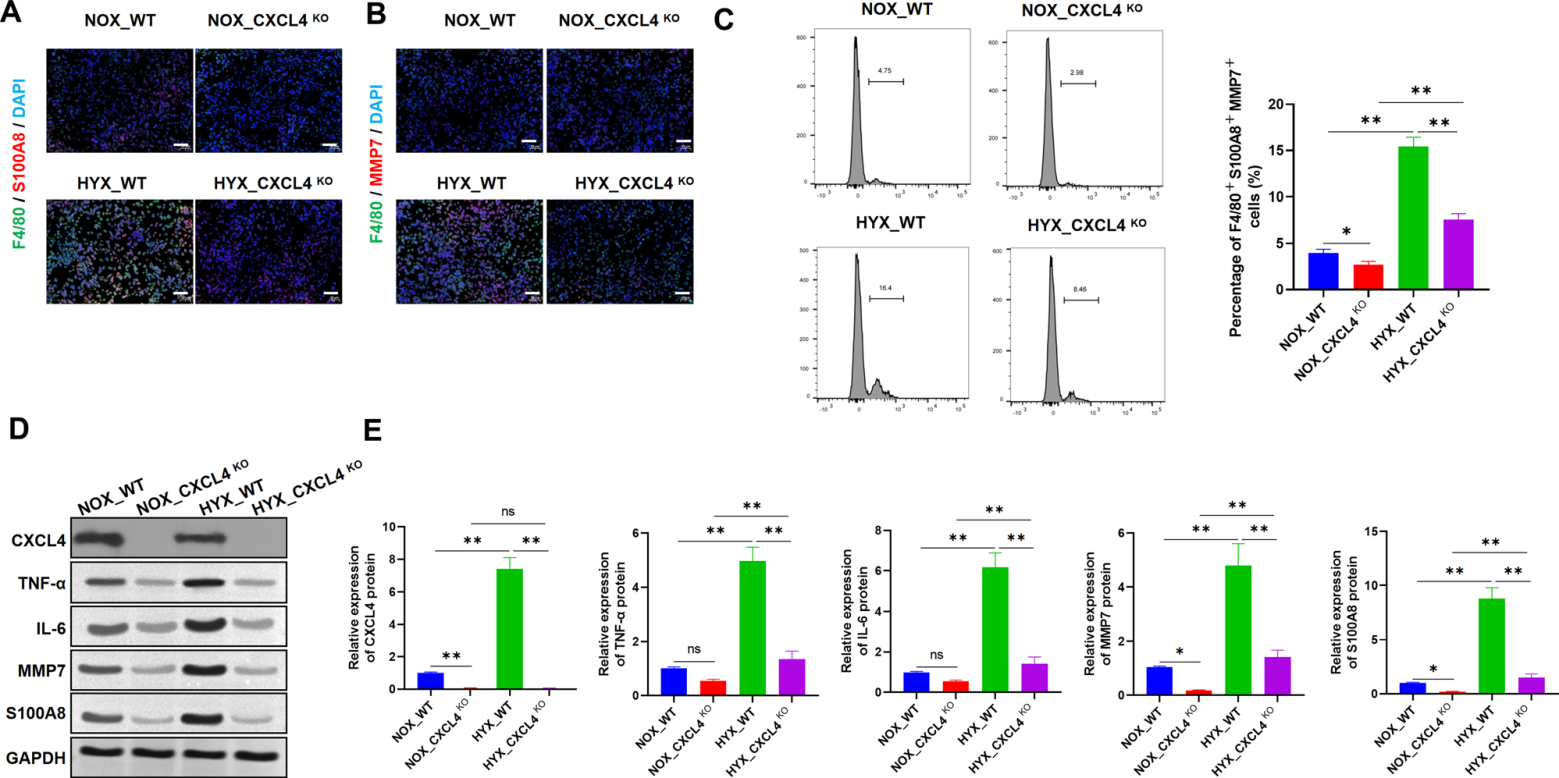
Deletion of CXCL4 prevents the progression of M4 macrophages in the lung. (**A**) IF staining for F4/80, S100A8, and DAPI in lung tissues from WT and CXCL4 KO mice under NOX (21% O_2_) and HYX (95% O_2_) conditions (*n* = 6). (**B**) IF staining for F4/80, MMP7, and DAPI (*n* = 6). (**C**) Flow cytometry analysis quantifying F4/80 + S100A8 + MMP7 + cells (%) (*n* = 6). (**D**-**E**) Western blot analysis of CXCL4, TNF-α, IL-6, MMP7, S100A8, and GAPDH (*n* = 6)

IF staining revealed elevated levels of F4/80 and S100A8 in HYX_WT mice compared to NOX_WT mice, while F4/80 and S100A8 expression was reduced in HYX_CXCL4 KO mice relative to HYX_WT mice (Fig. 4A). A similar pattern was observed for F4/80 and MMP7, with increased expression in HYX_WT mice compared to NOX_WT mice, and was relatively decreased expression in HYX_CXCL4 KO mice relative to HYX_WT mice (Fig. 4B). Flow cytometry analysis confirmed an increase in F4/80 + S100A8 + MMP7 + cells in HYX_WT mice compared to NOX_WT mice; with a relatively reduction in these cells in HYX_CXCL4 KO mice compared to HYX_WT mice compared to HYX_WT mice. Notably, the F4/80 + S100A8 + MMP7 + cell population in HYX_CXCL4 KO mice was higher than in NOX_CXCL4 KO mice (Fig. 4C). Western blot analysis indicated increased levels of CXCL4, TNF-α, IL-6, MMP7, and S100A8 proteins in HYX_WT mice compared to NOX_WT mice, while these protein levels were relatively reduced in HYX_CXCL4 KO mice. Moreover, the levels of CXCL4, TNF-α, IL-6, MMP7, and S100A8 proteins in HYX_CXCL4 KO mice were still higher than in NOX_CXCL4 KO mice (Fig. 4D-E). These results suggest that CXCL4 deficiency inhibits the expansion of M4 macrophages in the lungs under hyperoxic conditions.

### CXCL4 induces M4 macrophages in vitro and drives macrophage migration via chemokine (C-C motif) receptor 1

**Fig. 5:**
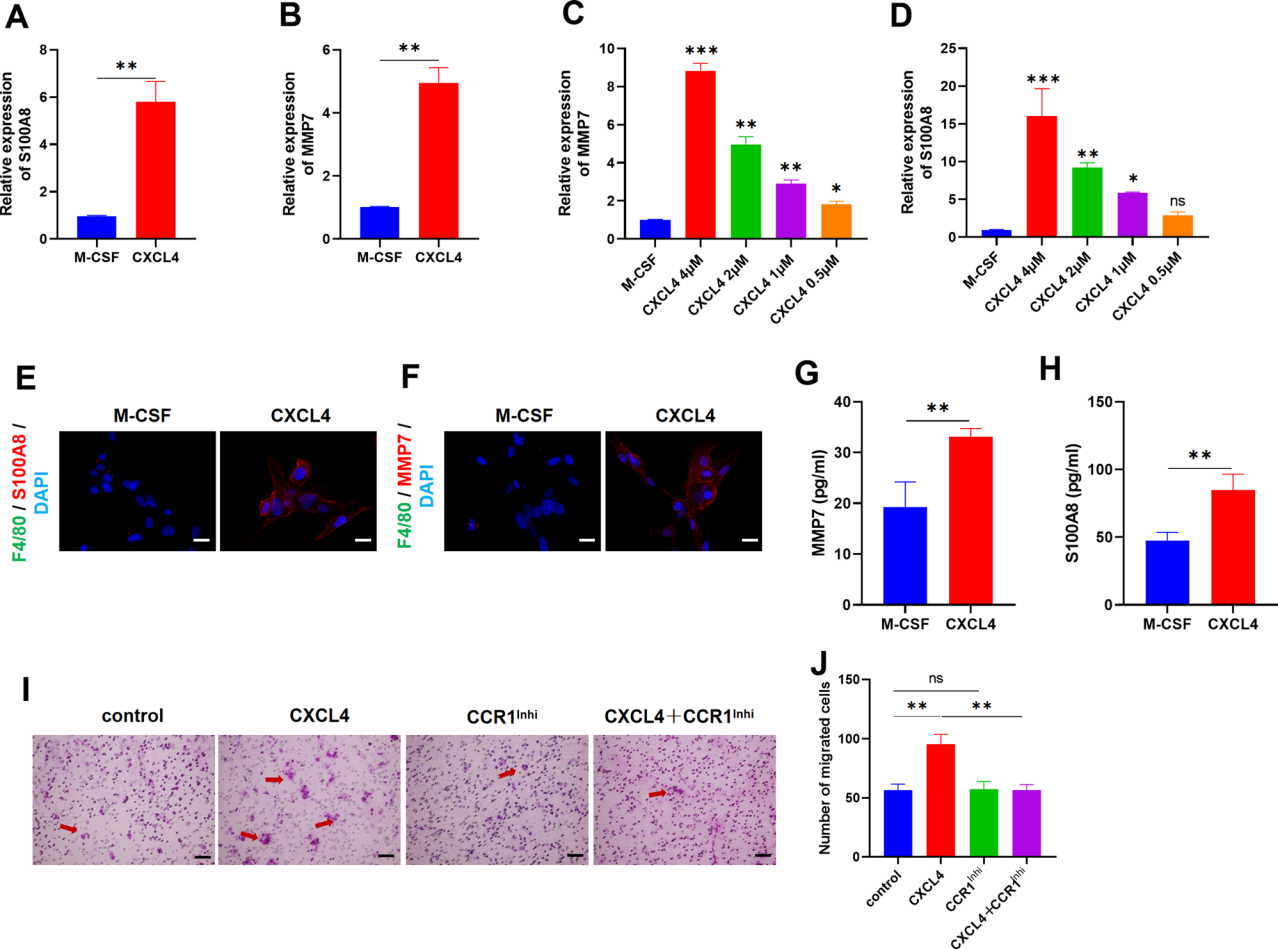
CXCL4 induces M4 macrophages in vitro and drives macrophage migration via CCR 1. (**A**-**B**) Expression levels of S100A8 and MMP7 in macrophages treated with M-CSF (M0) and CXCL4 (M4) (*n* = 3). (**C**-**D**) Dose-dependent expression of MMP7 and S100A8 in response to varying concentrations of CXCL4 (4, 2, 1, 0.5 µM) (*n* = 3). (**E**) IF staining of S100A8 and DAPI (*n* = 3). (**F**) IF staining of MMP7 and DAPI (*n* = 3). (**G**-**H**) ELISA results for MMP7 and S100A8 (*n* = 3). (**I**-**J**) Macrophage migration assay comparing control, CXCL4, CCR1 inhibitor (CCR1 Inhi), and CXCL4 + CCR1 Inhi groups. Arrows represented the type of migrated cells (*n* = 3)

Murine primary macrophages were treated with either M-CSF (100 ng/ml, denoted as M0) or CXCL4 (4 µM, denoted as M4). The expression of S100A8 and MMP7, markers of macrophage activation, was significantly upregulated in the CXCL4-treated group (Fig. 5A-B). Notably, their expression exhibited a dose-dependent response to CXCL4 (Fig. 5C-D), with concentrations comparable to those reported by Gleissner et al. (Gleissner et al. 2010). IF staining confirmed these results, revealing elevated S100A8 and MMP7 expression in CXCL4-treated macrophages (Fig. 5E-F). ELISA further corroborated the increase in MMP7 and S100A8 levels in the CXCL4 group (Fig. 5G-H). Moreover, CXCL4 enhanced macrophage migration, an effect that was reversed by a chemokine (C-C motif) receptor 1 (CCR1) inhibitor, indicating that CXCL4-induced macrophage migration partially relies on CCR1 signaling (Fig. 5I-J). Collectively, these data highlight the role of CXCL4 in modulating macrophage phenotype and function.

### CXCL4 deficiency promotes lung matrix remodeling during regeneration

**Fig. 6:**
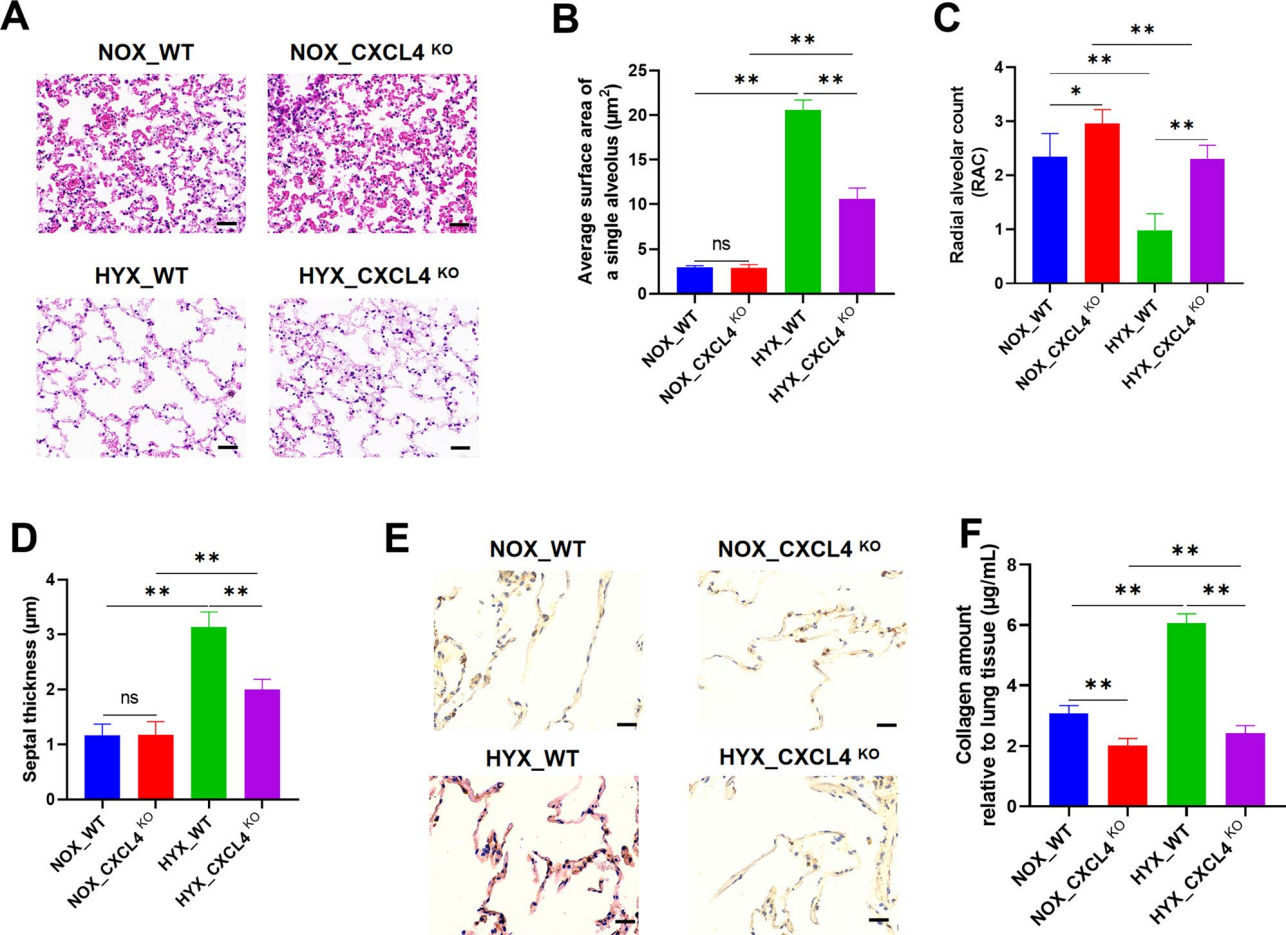
CXCL4 deficiency promotes lung matrix remodeling during regeneration. (**A**) H&E staining of lung tissues from WT and CXCL4 KO mice exposed to HYX followed by a recovery period under NOX conditions (*n* = 6). (**B**) Measurement of the average surface area of a single alveolus (µm²) (*n* = 6). (**C**) Radial Alveolar Count (RAC) analysis (*n* = 6). (**D**) Measurement of septal thickness (µm) (*n* = 6). (**E**-**F**) Quantification of collagen amount relative to lung tissue (*n* = 6)

Mice were exposed to hyperoxia to induce BPD, followed by 14 days of recovery in normoxic conditions. H&E staining revealed significant alterations in lung architecture in the HYX_WT group compared to the NOX_WT group, indicating injury and fibrotic remodeling. In contrast, the HYX_CXCL4 KO group exhibited notable improvements in lung architecture relative to the HYX_WT group (Fig. 6A). The average surface area was increased in HYX_WT mice compared to NOX_WT mice, but was relatively reduced in HYX_CXCL4 KO mice compared to HYX_WT mice. However, the surface area in HYX_CXCL4 KO mice was higher than in NOX_CXCL4 KO mice (Fig. 6B). The RAC was lower in HYX_WT mice but was relatively higher in HYX_CXCL4 KO mice, though still lower than in NOX_CXCL4 KO mice (Fig. 6C). Septal thickness was increased in HYX_WT mice relative to NOX_WT mice, but reduced in HYX_CXCL4 KO mice compared to HYX_WT mice. However, the septal thickness in HYX_CXCL4 KO mice remained higher than in NOX_CXCL4 KO mice (Fig. 6D). Finally, collagen content was elevated in HYX_WT mice compared to NOX_WT mice, but was relatively decreased in HYX_CXCL4 KO mice compared to HYX_WT mice. The collagen content in HYX_CXCL4 KO mice was still higher than in NOX_CXCL4 KO mice (Fig. 6E-F). These results suggest that CXCL4 deficiency enhances lung matrix remodeling during recovery from hyperoxia-induced injury.

## Discussion

Previous research has emphasized the significant involvement of CXCL4 in the development and management of lung diseases (Guo et al. 2017). The present study revealed the impact of CXCL4 on lung injury and regeneration under hyperoxic conditions, with a particular focus on its influence on M4 macrophages. In vivo, CXCL4 deletion mitigated hyperoxia-induced lung damage and promoted lung matrix remodeling in neonatal mice with BPD by restoring alveolar architecture and diminishing interstitial fibrosis. Moreover, CXCL4 deletion reduced M4 macrophage production in neonatal BPD models. In vitro, CXCL4 was shown to induce M4 macrophage formation and enhance macrophage migration through CCR1 regulation. Targeting CXCL4 and reducing its expression may offer therapeutic benefits for BPD.

Early-stage BPD is characterized by an inflammatory response and cytokine dysregulation (Leroy et al. 2018), marked by elevated proinflammatory cytokines, including IL-6, IL-8, and TNF-α, alongside reduced levels of anti-inflammatory cytokines such as IL-10 (Holzfurtner et al. 2022). CXCL4, a prominent proinflammatory chemokine in platelets, belongs to the CXC chemokine family but lacks the ELR sequence required for binding to CXCRs on neutrophils (Leiter et al. 2023). Experimental data suggest that CXCL4 modulates neutrophil recruitment and tissue injury in complex inflammatory conditions, such as lung fibrosis (Affandi et al. 2022). Additionally, Volkmann et al. reported significantly higher CXCL4 levels in systemic sclerosis patients with interstitial lung disease compared to healthy controls (Volkmann et al. 2016). Spaks et al. highlighted that elevated CXCL4 levels in non-small cell lung cancer patients correlated with poorer overall and disease-free survival (Spaks et al. 2016). Notably, numerous studies have demonstrated that hypoxia induces CXCL4 expression in various cell types (Ottria et al. 2022; Korbecki et al. 2021). Consistent with this, our study revealed increased CXCL4 expression in the HYX group compared to the NOX group. In contrast, a previous study in infants, with a BPD prevalence of up to 80.4% over more than 6 years, indicated that low serum CXCL4 levels in premature infants one week after birth were linked to a higher risk and severity of BPD (Arjaans et al. 2020). The observed discrepancy may be attributed to individual variability and differing diagnostic criteria.

Intriguingly, CXCL4 modulates monocyte differentiation by promoting the acquisition of macrophage-specific phenotypes, which has been designated as the “M4” phenotype (Domschke and Gleissner 2019). In systemic sclerosis, CXCL4 expression is upregulated in the lungs of both animal models of the disease, accompanied by increased M4 marker levels, and CXCL4 is involved in the alteration of phagocytic function of macrophages in this context (Tallec et al. 2024). In atherosclerosis, CXCL4 drives the differentiation of M4 macrophages, which co-express CD68, MMP7, and S100A8 (Erbel et al. 2015). Consistent with these observations, our study demonstrated that CXCL4 deletion mitigated lung structural damage and reduced M4 macrophage infiltration induced by hyperoxia in mice, supporting its protective role in hyperoxia-induced lung injury.

SPHK1 is an intracellular lipid enzyme that regulates cellular lipid metabolism, catalyzing the synthesis of S1P, and directly participates in cellular signal transduction (Ji et al. 2024). S1P produced intracellularly can be exported to the extracellular space via membrane transporters, where it binds to its receptor to indirectly activate intracellular signaling pathways (Li et al. 2020). The SPHK1/S1P axis modulates various cellular processes, including proliferation, apoptosis, migration, and inflammation (Jiang and Gong 2023). Accumulating evidence indicates a critical role of S1P metabolism in oxidative lung injury and the pathogenesis of BPD (Thomas et al. 2022). This study demonstrates that CXCL4 deficiency mitigates the downregulation of S1P metabolism-related genes induced by hyperoxia. In agreement, previous research has also implicated CXCL4 in modulating the inflammatory response and lipid metabolism (Gleissner et al. 2010).

Hyperoxia activates macrophages in immature lungs, disrupts alveolar type 2 cell homeostasis, and impairs elastic fiber formation, thereby inhibiting lung growth (Hirani et al. 2022). This study showed that CXCL4 deficiency mitigated hyperoxia-induced type 2 alveolar cell injury. Furthermore, CXCL4 deletion promoted the recovery of lung tissue and alveolar structures during the regenerative phase following hyperoxia-induced lung injury.

High-concentration oxygen exposure and prolonged inhalation of oxygen can trigger inflammation and acute lung injury, which is subsequently followed by fibrotic proliferation and repair, ultimately leading to pulmonary fibrosis. BPD is characterized by airway damage, inflammation, and pulmonary fibrosis (Ma et al. 2023; Zhu et al. 2024). In the present study, CXCL4 deletion was shown to reduce TNF-α and IL-6 levels, as well as M4 macrophage infiltration in response to hyperoxia exposure in vivo. In vitro, CXCL4 induced a dose-dependent polarization of macrophages toward the M4 phenotype. Moreover, CXCL4 promoted macrophage migration, an effect that was blocked by the CCR1 inhibitor. As a receptor for CXCL4, CCR1 mediates monocyte chemotaxis (Schwartzkopff et al. 2012) and is closely implicated in fibrosis (Affandi et al. 2022; Lande et al. 2020). Furthermore, CXCL4 deficiency led to reduced collagen deposition during the regenerative phase in newborn mice following hyperoxia exposure. Collectively, these results suggest that CXCL4 regulates M4 macrophages to modulate fibrotic lung remodeling during regeneration, potentially through the CCR1 signaling pathway.

Nonetheless, this study has several limitations. First, the expression of CXCL4 in human samples was not assessed. Second, clinical symptoms and the impact of CXCL4 on lung function in BPD mice were not investigated. Third, only male mice were used in the experiments. Furthermore, an in vivo model incorporating exogenous CXCL4 or a CXCL4 inhibitor in WT mice to examine CXCL4’s role in M4 polarization and its effects on BPD-related outcomes, such as pulmonary fibrosis, was not employed. Future research will address these aspects in greater detail.

## Conclusion

This study investigates the role of CXCL4 in the development of BPD. CXCL4 contributes to hyperoxia-induced lung injury and fibrotic remodeling by modulating cytokine secretion, alveolar cell proliferation, lipid metabolism, and macrophage phenotype and function. These results deepen our understanding of the molecular mechanisms underlying BPD and position CXCL4 as a potential therapeutic target for the condition.

## Data Availability

No datasets were generated or analysed during the current study.
